# Air Pollution Exposure and Gestational Diabetes Mellitus Risk: A Retrospective Case–Control Study with Multi-Pollutant Analysis in Wuhan, Hubei Province

**DOI:** 10.3390/toxics13020141

**Published:** 2025-02-19

**Authors:** Mengyang Dai, Jianfeng Liu, Min Hu, Feng Zhang, Yanjun Wang, Fangfang Dai, Rui Qu, Zhixiang Fang, Jing Yang

**Affiliations:** 1 Reproductive Medicine Center, Renmin Hospital of Wuhan University, Wuhan 430060, China; ddmmyy@whu.edu.cn (M.D.); zhangf@whu.edu.cn (F.Z.); wyj0108162025@163.com (Y.W.); daiff0515@whu.edu.cn (F.D.); qr200111@163.com (R.Q.); 2 The State Key Laboratory of Information Engineering in Surveying, Mapping and Remote Sensing, Wuhan University, Wuhan 430072, China; jianfengliu@whu.edu.cn; 3 Department of Gynaecology and Obstetrics, Renmin Hospital of Wuhan University, Wuhan 430060, China; huminmmmmm@whu.edu.cn

**Keywords:** air pollution, nitrogen dioxide, gestational diabetes mellitus, pregnancy trimester, case–control study

## Abstract

Ambient air pollution has been associated with gestational diabetes mellitus (GDM); however, evidence regarding trimester-specific effects from China remains limited. This case–control study study analyzed data from pregnant women who delivered in Wuhan, China, between 2017 and 2022 (164 GDM cases and 731 controls), integrating geographic information, air quality measurements, and maternal characteristics. Using Inverse Distance Weighting interpolation and Generalized Linear Mixed Models (GLMM), we assessed associations between air pollutant exposure and GDM across different gestational periods. Results indicated that NO_2_ demonstrated the strongest association with GDM compared to other pollutants. Specifically, increased NO_2_ exposure was consistently associated with higher GDM risk throughout pregnancy. PM_2.5_ exposure showed significant associations during early and mid-pregnancy, while SO_2_ exposure was significantly associated with GDM risk exclusively in early pregnancy. Sensitivity analyses stratified by urban maternity status and maternal age revealed the stability of the study’s findings. These findings underscore the importance of reducing air pollution exposure during pregnancy and implementing targeted interventions for high-risk populations to prevent GDM development.

## 1. Introduction

Gestational diabetes mellitus (GDM), a common obstetric complication characterized by glucose intolerance or hyperglycemia first recognized during pregnancy in women without pre-existing diabetes, is a metabolic condition that typically resolves postpartum [1]. The global prevalence of GDM exhibits considerable variation, differing significantly across regions, races, ethnicities, socioeconomic statuses, and diagnostic criteria [2]. A meta-analysis applying the International Association of Diabetes and Pregnancy Study Groups (IADPSG) criteria reported the overall incidence of GDM in mainland China to be 14.8% [3]. In recent decades, the prevalence of GDM has shown a significant upward trend globally, likely reflecting a complex interplay of factors including rising rates of overweight and obesity, advanced maternal age, growth of minority populations, and shifts in socioeconomic status and physical activity patterns among women of reproductive age [4]. In China specifically, the implementation of the universal two-child policy has been associated with an increase in the proportion of older pregnant women, which may serve as an independent risk factor contributing to the rising incidence of GDM [5].

GDM affects both maternal and fetal health. GDM independently increased the risk of composite adverse maternal outcome, caesarean delivery, pregnancy-induced hypertension, premature rupture of membranes, antepartum hemorrhage, and postpartum hemorrhage [6]. Women with a history of GDM are not only at an increased risk of developing type 2 diabetes mellitus (T2DM) but also exhibit elevated cardiovascular morbidity in later life, which is directly associated with an increased risk of cardiovascular disease-related mortality [7,8,9]. For the fetus, GDM increases the risk of adverse pregnancy outcomes such as preterm birth, macrosomia, and neonatal respiratory distress syndrome [10]. Furthermore, offspring exposed to GDM in utero may face an increased risk of various long-term health complications, including childhood obesity, autism spectrum disorders, ophthalmic diseases, and glucose intolerance [11,12,13,14].

In addition to traditional demographic and clinical risk factors for GDM, including advanced maternal age (>35 years), being overweight or obese, family history of type 2 diabetes, previous history of GDM, previous macrosomic infant (>4 kg), previous intrauterine death, hypertension, polycystic ovary syndrome (PCOS), multiparity, and certain ethnic origins, growing evidence suggests that environmental factors, particularly air pollution, may play a significant role in the development of GDM [15,16,17]. Longitudinal studies have provided compelling evidence that long-term exposure to air pollutants can contribute to the development of insulin resistance, a key mechanism in GDM pathogenesis. For example, research has shown that per the interquartile range increment in annual average air pollutant concentrations, insulin resistance markers significantly increased by 2.5% for coarse particulate matter, 3.1% for ${\mathrm{PM}}_{2.5}$, and 3.2% for nitrogen dioxide [18]. Studies have demonstrated various environmental chemical exposures can affect blood glucose levels during pregnancy: exposure to certain perfluoroalkyl substances (PFASs) was associated with elevated blood glucose levels in Chinese pregnant women [19], while environmental exposure to polycyclic aromatic hydrocarbons and phthalate acid esters (PAHs and PAEs) was positively associated with gestational glucose levels and GDM risks [20]. Air pollution exposure, particularly to fine particulate matter (PM_2.5_), has been shown to induce endothelial dysfunction and contribute to insulin resistance, potentially serving as a mechanistic link between environmental factors and GDM pathogenesis. Indeed, ambient air pollution has emerged as a serious global environmental concern, with nearly 99% of the world’s population exposed to air that exceeds WHO quality limits, and this issue is particularly pronounced in China where rapid industrialization has led to widespread air quality standard violations, potentially contributing to various adverse pregnancy outcomes including gestational diabetes mellitus (GDM), low birth weight (LBW), preterm birth (PTB), and hypertensive disorders of pregnancy (HDP) [21,22,23].

GDM’s well-established risk factors alone cannot fully explain its rapid increase worldwide in recent decades, suggesting the potential role of environmental factors in its development. Recent meta-analyses have demonstrated that exposure to air pollutants significantly increases GDM risk, with estimated odds ratios of 1.09 (95% CI: 1.06–1.12) for ${\mathrm{PM}}_{2.5}$ and 1.17 (95% CI: 1.04–1.32) for ${\mathrm{PM}}_{10}$ [24]. Epidemiological studies have shown that ambient air pollution exposure is directly associated with higher fasting blood glucose and 1-hour glucose concentrations during pregnancy [25]. Mechanistically, air pollution exposure may increase insulin resistance and incite metabolic dysfunction through multiple pathways [18]. These include accelerated β-cell dysfunction in susceptible pregnant women, altered adipokine concentrations (particularly leptin and adiponectin) affecting neurohormonal metabolic control, and increased local or systemic inflammatory responses [26]. Furthermore, components of air pollutants, such as metals, sulfur, and organic compounds, may stimulate oxidative damage through ROS production, affect glucose transport, and trigger inflammatory responses that impact insulin signaling [27].

The present study aimed to investigate the associations between exposure to various air pollutants (including NO_2_, PM_2.5_, SO_2_, O_3_, CO, and PM_10_) and the risk of gestational diabetes mellitus across different pregnancy stages. To assess the robustness of our findings, we conducted sensitivity analyses examining whether these associations remained consistent across different urbanization levels and maternal age groups. By elucidating the temporal patterns of air pollution exposure in relation to GDM risk and identifying potentially susceptible populations, this study endeavors to provide evidence-based insights for developing targeted preventive strategies and interventions to reduce the burden of GDM among pregnant women exposed to air pollution.

## 2. Method

### 2.1. Study Design and Population

We conducted a hospital-based case–control study to investigate the associations between air pollution exposure and gestational diabetes mellitus (GDM). The study design consisted of three key components: (1) case–control selection through strict inclusion and exclusion criteria, (2) assessment of air pollution exposure across different pregnancy stages, and (3) analysis of associations while controlling for potential confounders. Both cases (pregnant women diagnosed with GDM according to IADPSG criteria) and controls (pregnant women without GDM) were selected from the same hospital population during the same period, following identical inclusion and exclusion criteria. The robustness of our findings was further validated through sensitivity analyses stratified by urban maternity status and advanced maternal age. In this study, information on pregnant women who gave birth in Wuhan University People’s Hospital from 1 September 2017 to 30 September 2022 was counted. Maternal demographic information (e.g., maternal age, education level, occupation, household registration, gestation time, gestational age), pregnancy outcome, and infant information (e.g., infant sex, birth weight, delivery time, litter size) were collected. We excluded pregnant women who were not eligible for the study according to the following exclusion criteria:

1. Women with maternal comorbidities of thrombocytopenia, severe anemia, hyperemesis gravidarum, diabetes mellitus, abnormal thyroid function, inflammatory diseases, or liver disease;
2. Women with obstetric complications of placental implantation, placenta previa, and intrauterine distress;
3. Women who lacked data on exposure to atmospheric pollution prior to pregnancy and lacked matching of street addresses or distances to the monitoring site information;
4. Women with multiple pregnancies;
5. Pregnant women with gestational weeks less than 37 weeks or greater than 42 weeks;
6. Women with less than 2 years of residence in their registered place of residence;
7. Stillbirths;
8. Missing characteristic data in any of the following categories: maternal demographic characteristics, pregnancy outcomes and infant characteristics.

After applying the exclusion criteria, 164 women diagnosed with gestational diabetes mellitus (GDM) were selected as the case group, while 731 women without GDM were randomly selected as the control group (total n = 895). The sample size in this study was determined by the available data from the hospital’s maternal health monitoring system during the specified time period, rather than being calculated a priori. A post-hoc power analysis was conducted to assess the statistical power of the achieved sample size. With α = 0.05, and assuming a medium effect size (Cohen’s d = 0.3) for air pollution exposure effects, our study achieved sufficient power to detect meaningful associations between air pollutant exposure and GDM risk. GLMM was used to determine the impact of pollutants in different groups and periods. All personal information had already been deidentified to protect patients’ privacy. This study was approved by the Ethics Committee of Wuhan University Renmin Hospital, Hubei Province, China (The ethical approval number: WDRY2023-K038).

### 2.2. Study Area

Wuhan is situated in eastern Hubei Province within the middle reaches of the Yangtze River (113°$41^{\prime }$–115°$05^{\prime }$ E, 29°$58^{\prime }$–31°$22^{\prime }$ N). The city, intersected by the Yangtze River and Han River, encompasses a total area of 8569.15 square kilometers and is characterized by a subtropical monsoon humid climate. As of the end of 2022, the city’s permanent resident population reached 13.739 million. Located in the Jianghan Plain, Wuhan experiences distinctive seasonal variations with hot, humid summers and cold, dry winters, exhibiting significant temperature fluctuations. Due to its numerous waterways, convenient transportation, and advantageous geographical location, it serves as a crucial hub in China. Rapid urbanization, high population growth, large number of vehicles, and high energy consumption have all led to substantial environmental pressures. The city’s air quality exhibits distinct seasonal patterns, with PM_2.5_, PM_10_, SO_2_, NO_2_, and CO concentrations peaking in winter and reaching their lowest levels in summer, while O_3_ shows an opposite trend with maximum levels in summer. Spatial distribution analysis reveals higher concentrations of particulate matter in urban and industrial areas, elevated SO_2_ levels in industrial zones, and higher O_3_ levels in suburban areas. These pollution patterns are strongly influenced by meteorological conditions, with temperature and precipitation playing crucial roles in pollutant dispersion [28].

### 2.3. Diagnostic Criteria

According to IADPSG criteria, GDM is diagnosed if one or more of the following plasma glucose values in the 75 g OGTT are met or exceeded:

- Fasting plasma glucose (FPG) ≥ 5.1 mmol/L (92 mg/dL);
- 1-hour plasma glucose ≥ 10.0 mmol/L (180 mg/dL);
- 2-hour plasma glucose ≥ 8.5 mmol/L (153 mg/dL).

### 2.4. Exposure Assessment

**Figure 1:**
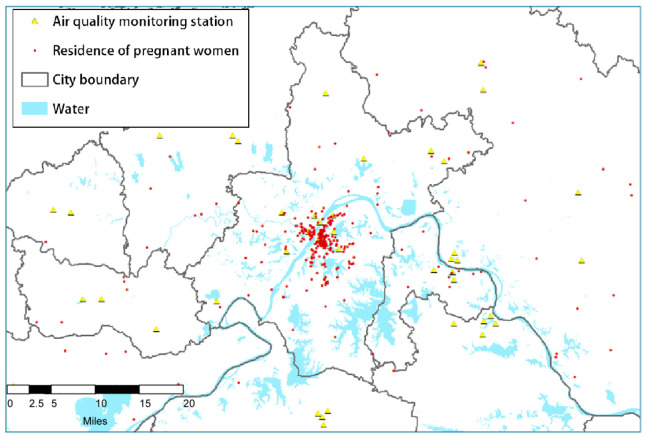
Distribution of monitoring stations and cases.

This study utilized the residential addresses of pregnant women to assess their daily exposure to air pollutants. The geographical coordinates (latitude and longitude) of these residences were acquired through forward geocoding via the Baidu Maps API, as illustrated in Figure 1. Daily average air quality data were sourced from the National Meteorological Administration of China, accessible at http://data.cma.cn/, accessed on 3 January 2025. In accordance with the national standard operating procedures for air quality monitoring (http://data.cma.cn/, accessed on 3 January 2025), gaseous pollutant concentrations were converted into micrograms per cubic meter (μg/m^3^) to facilitate comparisons with existing research. Continuous measurements of air quality concentrations were obtained from 435 fixed national control air quality monitoring stations situated throughout the country. Any gaps in the data were addressed using the bootstrap method [29]. Figure 1 depicts the locations of both the air monitoring stations and the case study sites, with red dots representing the case points and blue triangular dots indicating the positions of the air quality monitoring stations.

(1) AP_station|(xs,ys,T)={COs,SO2s,NO2s,O3s,PM2.5s,PM10},

(2) AP_case|(X,Y,T)={COc,SO2c,NO2c,O3c,PM2.5c,PM10c},

(3) $$d_{i}=\sqrt{{\left(X-xs_{i}\right)}^{2}+{\left(Y-ys_{i}\right)}^{2}},$$

(4) $$w_{i}=\frac{\frac{1}{d_{i}}}{\sum_{1}^{n}\frac{1}{d_{i}}},$$

(5) $$AP_{c}=\sum_{i=1}^{i=n}w_{i}*AP_{s}\left(xs_{i},ys_{i},T\right).$$

During the study period, the Inverse Distance Weighting (IDW) algorithm was employed to estimate the natural ambient exposure levels at each participant’s residential address, as outlined in equations [30]. In these equations, AP_station denoted the concentration of air pollutants at the monitoring station, while AP_case represented the exposure concentration at the case location. The coordinates xs and ys indicated the position of the monitoring station, whereas X and Y represented the coordinates of the case location. The Euclidean distance from the case point to the monitoring station was denoted by di, and wi represented the weight assigned in the IDW algorithm.Through cross-validation experiments, we determined an optimal power parameter of 3 for the IDW interpolation, achieving an R^2^ of 0.78. Consequently, AP_case was calculated using the IDW approach.

We processed the aforementioned data into a raster format and extracted the eigenvalue at each case location to serve as independent variables for subsequent analysis. All of these processing steps were executed in ArcGIS 10.7. We calculated the average exposure levels of CO, SO_2_, NO_2_, O_3_, PM_2.5_, and PM_10_ for each participant across four distinct periods:

- all period: the entire period of pregnancy (last menstruation to delivery);
- period 1: the first trimester (last menstruation to third month);
- period 2: the second trimester (third month to sixth month);
- period 3: the third trimester (sixth month to delivery).

These calculations were performed to assess the maternal exposure to air pollutants.

### 2.5. Statistical Analysis

We decided to employ the GLMM as our primary analytical tool to thoroughly explore the impact patterns of air pollution on GDM across diverse populations. Air pollutant exposures (NO_2_, PM_2.5_, SO_2_, O_3_, CO, and PM_10_) were analyzed as continuous variables to preserve their original distribution characteristics and enable examination of exposure-response relationships. Our analytical approach progressed systematically from single-pollutant to multi-pollutant models. In single-pollutant models, we assessed the individual effects of each pollutant. Before developing multi-pollutant models, we diagnosed multicollinearity using variance inflation factor (VIF), with pollutants showing high correlations (VIF > 10) being selectively included in the final models. The relative impacts of different pollutants were compared using standardized regression coefficients. The regression coefficients obtained in multi-pollutant models were interpreted as independent effects after controlling for other pollutants, allowing us to effectively isolate the specific impact of each air pollutant on GDM risk. Model stability was evaluated through extensive sensitivity analyses, including stratification by urban/rural status and maternal age to assess the consistency of associations across different population subgroups. This model allows us to account for both fixed and random effects, which is crucial when dealing with complex data structures that often characterize environmental health studies.

To further delve into the differences in biological and sociological factors among the cases in our study, we utilized the $\chi^{2}$ test. This statistical method enabled us to rigorously assess variations in the effects of various covariates, such as age, ethnicity, and socioeconomic status, on the outcomes of GDM. By doing so, we aimed to identify any potential confounding factors that might influence the relationship between air pollution exposure and GDM risk.

In addition to the statistical analyses, we also compiled detailed descriptive statistics for pollutant exposure during different periods of pregnancy. This included calculating the mean, median, and range of exposure levels for key air pollutants like NO_2_, PM_2.5_, PM_10_, SO_2_, and O_3_ during each trimester. These statistics provided us with valuable insights into the exposure levels that pregnant women in our study population were experiencing, allowing us to better understand the potential risks associated with different levels of air pollution exposure during various stages of pregnancy.

We adopted Odds Ratios (OR) to describe changes in the risk of GDM prevalence. Results were expressed as changes in the risk of GDM prevalence per unit increase in each pollutant. All statistical analyses were executed using SPSS version 22.0, with statistical significance determined at a two-sided significance level (p<0.05).

### 2.6. Covariates

Maternal age, maternal education, type of residence, family income, gravidity, parity, pre-pregnancy BMI, conception method, gestational age at birth, mode of delivery, infant sex, and season of pregnancy were included as covariates. We classified the covariates according to the following criteria:

1. Maternal age was classified as 24 years and below, 25–29 years, 30–34 years, 35 years and above;
2. Type of residence was classified as urban, rural;
3. Education level was classified as university and above, college, high school and secondary school, junior high school and below;
4. Annual per capita income was classified as greater than 10 w, less than or equal to 10 w, and unspecified;
5. The number of pregnancies was divided into 1, 2, 3, 4 and above;
6. The number of deliveries was divided into 0, 1 and 2;
7. The pre-pregnancy BMI was divided into less than 18.5, greater than or equal to 18.5 and less than 24, greater than or equal to 24 and less than 28, greater than or equal to 28 and unclear;
8. The mode of conception was divided into assisted reproduction and natural conception;
9. The week of pregnancy was divided into less than 37 weeks, 37 weeks–42 week;
10. The mode of delivery were divided into vaginal delivery and cesarean.

## 3. Result

### 3.1. Characteristics of the Participants

A total of 895 pregnant women who fulfilled the study criteria participated in the study, 164 in the case group and 731 in the control group. Table 1 shows their basic physiological and sociological features. The mean age of all subjects was 31.63 years. The majority of mothers (80.45%) were between 25 and 34 years old, and for those with known BMI values (35.75% of total participants), 22.35% had a BMI between 18.5 and 23.9 kg/m^2^, 9.83% had a BMI > 24, and 3.58% had a BMI < 18.5. About 92.85% of pregnancies were natural conceptions, and 90.28% of mothers were supplemented with folic acid. Most mothers (64.92%) had a college degree or above, 91.06% had no history of smoking, and 97.88% had no infectious diseases. Furthermore, we found that GDM was not significantly associated with type of residence, maternal education, family income, gravidity, infectious disease, smoking status, folic acid supplementation, delivery method, and infant sex (p>0.05). However, GDM was significantly associated with maternal age (p<0.01), gestational age (p<0.05), risk stratification in pregnancy (p<0.01), BMI (p<0.01), natural conception (p<0.05), assisted reproductive technology (p<0.05), year (p<0.01), and season of delivery (p<0.01). The mothers who were older than 30 years, were classified as medium risk during pregnancy, and delivered in summer were at increased risk of GDM.

### 3.2. Characteristics of Ambient Air Pollutants

As shown in Table 2, PM_2.5_, which measures particulate matter with a diameter of 2.5 micrometers or less, we observe a wide range of concentrations. The minimum exposure is 6.04 μg/m^3^, while the maximum soars to 156.76 μg/m^3^. The median value of 33.57 μg/m^3^ suggests that half of the measurements fall below this level, and half above. The IQR of 24.45 μg/m^3^ indicates a significant variability in exposure levels between the 25th and 75th percentiles. The mean exposure of 20.12 μg/m^3^ is lower than the median, suggesting a skewed distribution towards lower values, yet still above the ideal levels recommended by AQG2021.

PM_10_, which measures particulate matter up to 10 micrometers in diameter, follows a similar pattern with higher concentrations compared to PM_2.5_. The minimum exposure is 13.08 μg/m^3^, and the maximum reaches a staggering 507.36 μg/m^3^. The median value of 65.88 μg/m^3^ and the mean of 45 μg/m^3^ indicate that exposures are generally higher than those for PM_2.5_, reflecting a more polluted environment.

For gaseous pollutants, SO_2_ levels range from a minimum of 2.23 μg/m^3^ to a maximum of 63.06 μg/m^3^, with a median of 7.68 μg/m^3^. The mean value of 8.30 μg/m^3^ suggests a relatively normal distribution. NO_2_ concentrations span from 5.75 μg/m^3^ to 82.73 μg/m^3^ with a median of 35.41 μg/m^3^ and a mean of 35.29 μg/m^3^, indicating higher overall exposure compared to SO_2_.

O_3_, measured as a single-day maximum eight-hour average, exhibits a minimum of 26.78 μg/m^3^, and a maximum of 210.88 μg/m^3^. The median value of 112.51 μg/m^3^ and the mean of 112.27 μg/m^3^ suggest that exposures are generally high, potentially posing health risks. CO levels, measured in milligrams per cubic meter, range from 0.14 μg/m^3^ to 2.67 μg/m^3^, with a median of 0.72 μg/m^3^ and a mean of 0.75 μg/m^3^. These values indicate that while some exposures are low, others are significantly higher, reflecting variable pollution levels.

**Figure 2:**
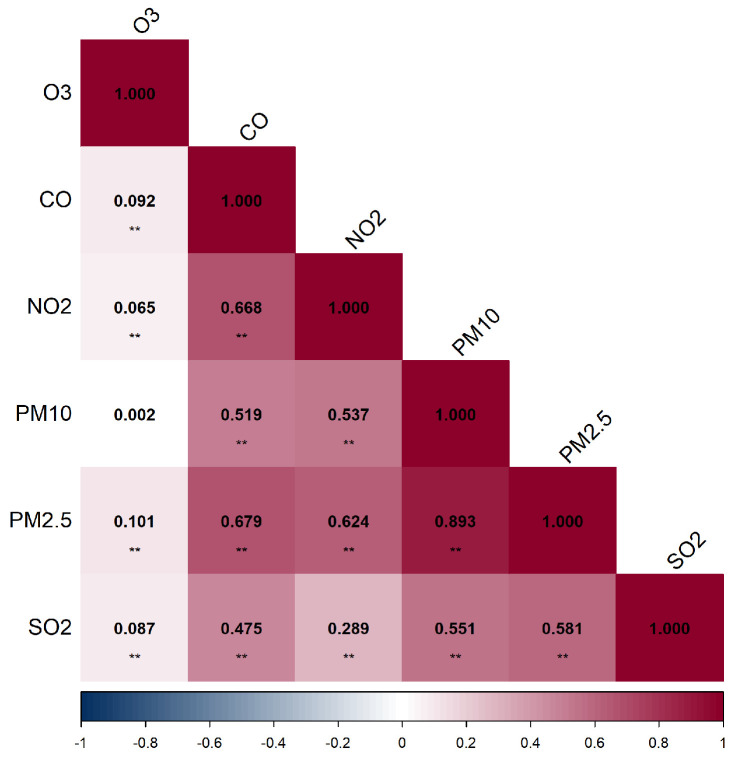
Pearson correlation coefficient between air pollutants, ** *p* < 0.01.

The IQR and SD values for all pollutants provide valuable insights into the variability and dispersion of exposure levels. For instance, the high IQR for PM_2.5_ (24.45 μg/m^3^) and PM_10_ (42.47 μg/m^3^) suggests substantial variability in exposure levels across different locations or time periods. Similarly, the SD values reflect the degree of spread around the mean, with higher SD indicating greater variability. During the entire pregnancy period, PM_2.5_ exposure showed very strong correlation with PM_10_ exposure (r=0.893, p<0.01). O_3_ exposure exhibited very weak correlations with all other pollutants, while moderate to strong correlations were observed among other pollutants (*r* ranging from 0.475 to 0.679, p<0.01) (Figure 2).

### 3.3. Association Between Air Pollution and the Risk of GDM

The analysis of air pollutants and GDM has revealed significant associations across different stages of pregnancy. Table 3 presents the ORs and corresponding *p*-values for the associations between GDM and exposure to various air pollutants, stratified by exposure windows including the first, second, and third trimesters, as well as the entire gestational period. NO_2_ consistently demonstrated a significant positive correlation with GDM throughout the entire pregnancy, with the strongest association observed in the third trimester (OR: 1.352, 95% CI: 1.067–1.637, p=0.020). This suggests that exposure to higher concentrations of NO_2_ during the third trimester significantly increases the risk of developing GDM.

Additionally, PM_2.5_ showed significant positive associations with GDM in both the first and second trimesters, with the most pronounced effect in the first trimester (OR: 1.082, 95% CI: 1.030–1.134, p=0.014). This indicates that exposure to PM_2.5_ during early pregnancy may also increase the risk of GDM. SO_2_ exhibited a significant positive association with GDM only in the first trimester (OR: 1.255, 95% CI: 1.059–1.452, p=0.010), highlighting the critical period of early pregnancy for the impact of SO_2_ on GDM. While O_3_ showed a significant association with GDM in the first trimester, CO and PM_10_ did not demonstrate statistically significant associations with GDM in any trimester. These findings suggest that exposure to specific air pollutants, particularly NO_2_, PM_2.5_, and SO_2_, may increase the risk of developing gestational diabetes mellitus, with varying effects across different stages of pregnancy.

Sensitivity analyses were conducted by stratifying the study population according to urban maternity status and advanced maternal age to examine the robustness of associations between air pollutants and GDM. As shown in Table 4, consistent positive associations were observed between GDM risk and exposure to SO_2_, NO_2_, and PM_2.5_. The associations for SO_2_ and NO_2_ appeared to be stronger among women in urban areas and those of advanced maternal age. These stratified analyses supported the stability of our main findings, while also suggesting potential effect modification by urbanization and maternal age.

## 4. Discussion

In this present study, we utilized a comprehensive dataset consisting air pollution data reproductive health study focused on GDM in China. Employing a multifaceted analytical approach that included integrated correlation analysis and linear mixed models, we aimed to identify the key natural environmental factors influencing changes in GDM risk and to quantify the extent of their impact on this condition. Our findings revealed that, among the various parameters related to GDM risk were particularly susceptible to the influence of gaseous pollutants, suggesting that environmental pollutants may play a significant role in modulating the risk of GDM. IDW interpolation in public health research offers several benefits. It enhances prediction accuracy by assigning weights based on distance, providing a detailed spatial distribution of health data. This facilitates visualization and further analysis, aiding in the formulation of targeted public health policies. IDW is also efficient for large datasets and versatile for various environmental data, ultimately supporting evidence-based decision-making.

In this comprehensive analysis of air pollutants and GDM, our findings reveal distinct temporal patterns of association across different pregnancy stages. Notably, NO_2_ emerged as the most consistently associated pollutant, demonstrating significant positive correlations throughout pregnancy: the association was pronounced in the first trimester, decreased slightly in the second trimester, and reached its peak in the third trimester. PM_2.5_ showed trimester-specific effects, with significant associations in both the first and second trimesters, particularly pronounced during early pregnancy. Similarly, SO_2_ exhibited a significant relationship with GDM risk, though this was confined to the first trimester. Although ozone exhibited a significant association during the first trimester, the pregnancy-averaged exposures of ozone, carbon monoxide, and PM_10_ showed no statistically significant associations across the entire gestational period.

Recent years have witnessed extensive research investigating the association between air pollution and GDM. Literature analysis demonstrates that the majority of studies have identified significant positive correlations between fine PM_2.5_ and GDM, with sensitive exposure windows spanning from early to late pregnancy stages. This finding aligns with our study’s observation of significant associations between PM_2.5_ exposure and GDM risk during early and mid-pregnancy periods. Notably, there exists a marked distinction between findings from developed and developing countries. Studies conducted in developed nations predominantly report associations between PM_2.5_ and GDM [31,32,33], whereas research from developing countries, particularly China, which faces more severe air pollution challenges, frequently identifies associations with multiple pollutant components. This divergence may be attributed to the generally higher air pollution levels prevalent in developing countries. Regional studies within China exhibit distinct geographical characteristics, particularly exemplified in South China’s Guangdong Province, where two independent cohort studies demonstrated remarkable consistency. Both studies from this southern region revealed that exposure to particulate matter (both PM_2.5_ and PM_10_) was positively associated with elevated fasting glucose levels during pregnancy [34,35]. Two studies from Northeast China consistently highlighted significant associations between GDM and gaseous pollutants, particularly NO_2_ and SO_2_, despite differences in exposure timing [36,37]. In Central China, our study demonstrated significant associations between PM_2.5_ and GDM risk during early and mid-pregnancy periods, with NO_2_ showing consistent associations throughout pregnancy and the strongest effect in the third trimester. Similarly, research from Hefei, a neighboring city, identified pre-pregnancy exposure to multiple pollutants (PM_2.5_, PM_10_, O_3_, and SO_2_) was associated with GDM development [38]. These findings not only underscore the ubiquitous nature of the association between air pollution and GDM but also reveal region-specific patterns of pollutant-health effects, providing crucial scientific evidence for developing targeted regional prevention strategies.

In the present study, we observed a consistent positive association between NO_2_ exposure and GDM risk throughout pregnancy, with relatively higher effects in the first trimester than the second trimester, and the strongest association manifesting in the third trimester. The temporal pattern of these associations merits careful consideration within the context of existing literature, which has yielded heterogeneous findings regarding critical exposure windows. A recent systematic review highlighted the first trimester as the most critical exposure window, reporting the strongest associations between NO_2_ exposure and GDM risk during this period [39]. This finding has been substantiated by multiple cohort studies, including two retrospective analyses in the United States [40,41] and a prospective investigation in Denmark [42]. Moreover, evidence from a retrospective cohort study in Northeast China demonstrated that pre-pregnancy NO_2_ exposure significantly increased GDM risk, with dietary patterns modifying this association [36], - a finding that aligned with observations from a California cohort study [43]. Additionally, a prospective cohort study from Northeast China identified the second trimester as the critical window [37].

Regional meteorological conditions and seasonal activities may contribute to these inconsistencies. Factors such as weak winter monsoons impeding NO_2_ dispersion and seasonal agricultural practices affecting NO_2_ concentrations highlight the importance of considering local environmental contexts in air pollution studies. Previous studies have paid limited attention to the analysis of pollutant exposure during late pregnancy. Although most guidelines recommend oral glucose tolerance test at 24–28 weeks of gestation [44], GDM is defined as glucose intolerance first detected or developed during pregnancy [45], suggesting that research on late pregnancy also holds significant importance. Additionally, placental hormones play a crucial role in the pathogenesis of GDM, with both Placental Growth Hormone (PGH) and human Placental Lactogen (hPL) being synthesized in syncytiotrophoblast cells (SCTB) and showing significant increases during mid-pregnancy [46]. Air pollution may affect placental development through multiple mechanisms. Studies have shown that NO_2_ exposure is associated with reduced placental weight [47], and prenatal air pollution exposure might lead to nitrosative stress and epigenetic alterations in the placenta [48]. The potential mediating effect of air pollution on GDM through placental dysfunction, combined with the possible cumulative physiological effects of pollutant exposure, collectively explains why the critical exposure window might extend into late pregnancy. Subgroup analyses revealed that advanced maternal age was associated with increased susceptibility to air pollution-induced GDM, which might be attributed to enhanced placental endocrine function and hormone secretion in older pregnant women.

NO_2_ is a chemical compound that belongs to a group of highly reactive gases called nitrogen oxides (NOx), like nitric oxide (NO). They are the major pollutants of the earth’s atmosphere. NO_2_ is seemed as mainly an outdoor air pollutant, anthropogenic NO_2_ emissions come mainly from internal combustion engines and power plants, but also from heating or electricity generation [49]. In urban China, indoor and outdoor sources contribute comparably to total NO_2_ exposure [50]. Wuhan merits particular attention due to its elevated NO_2_ burden, which is exacerbated by unfavorable winter meteorological conditions, particularly weak monsoons that impede pollutant dispersion. The seasonal intensification of NO_2_ pollution between October and March is further aggravated by regional agricultural activities [51,52]. In light of these substantial NO_2_ exposure patterns in Wuhan, enhanced GDM screening and exposure prevention measures warrant consideration for pregnant women during high-pollution winter months. To address these NO_2_ exposure concerns, comprehensive control strategies at multiple levels are essential. At the industrial level, implementation of low-NO_x_ burners and flue gas treatment technologies in power plants can effectively reduce emissions from stationary sources [49]. For mobile sources, strengthening traffic emission controls through clean energy vehicle promotion is crucial, particularly given the significant contribution of vehicle emissions to urban NO_2_ levels—recent research suggests that aggressive electric vehicle adoption could reduce NO_2_ concentrations by up to 1.9 ppb in communities near major roadways [53]. At the individual level, since both indoor and outdoor sources contribute to total exposure in urban China, a dual-focused protection strategy is recommended for pregnant women: outdoor exposure can be reduced by limiting activities during peak pollution periods, while indoor exposure can be mitigated through proper ventilation, the use of HEPA and carbon filters air purifiers, and considering electrical alternatives to gas appliances where feasible [54]. These targeted intervention measures are particularly critical during the winter months when NO_2_ pollution typically intensifies in Wuhan.

The underlying biological mechanisms linking air pollution to GDM likely operate through multiple interconnected pathways. The primary pathway involves pancreatic β-cell dysfunction, which is particularly significant during pregnancy when β-cell machinery deficiencies may already exist [55]. PM_2.5_ exposure can downregulate glucose transporter 2 expression in pancreatic β-cells [56], while O3 may damage β-cells through altered T-cell-dependent immune responses, leading to reduced insulin secretion [57]. These effects are compounded by air pollution’s impact on neurohormonal function, particularly through the disruption of adipokine balance. Studies have shown that PM_2.5_ and NO_2_ exposure can alter levels of leptin and adiponectin [58,59], crucial regulators of metabolic control, with prenatal NO_2_ exposure specifically linked to modified adipokine profiles in cord blood [60]. Furthermore, air pollutants trigger both local and systemic inflammation through various pathways, with PM_2.5_ particularly affecting visceral adipose tissue inflammation through CC-chemokine receptor 2-dependent and -independent pathways, producing proinflammatory mediators including TNF-α, IL-6, and IL-8 [61,62,63].

The pathogenic effects of air pollution are further amplified through oxidative stress mechanisms and alterations in gut microbiota. Pregnancy-related hyperglycemia combined with exposure to air pollutants, especially metal-enriched particulate matter and organic components, can enhance oxidative stress through multiple mechanisms, leading to decreased glucose transporter expression and activation of NF-κB-mediated inflammatory responses [64,65,66]. Recent evidence suggests that air pollution can also modify gut microbiota composition and increase gut permeability, promoting the movement of inflammatory mediators into circulation [67,68]. The collective impact of these pathways—including β-cell dysfunction, inflammation, oxidative stress, and gut microbiota alterations—creates a complex pathophysiological environment that promotes metabolic dysregulation. The timing of exposure appears crucial, with early pregnancy representing a particularly vulnerable window due to the establishment of maternal–placental–fetal metabolic adaptations [69]. These mechanisms might contribute to varying susceptibilities among different population subgroups, though further research is needed to fully understand the potential effect modifications by factors such as urbanization status and maternal age.

Several limitations of this study should be acknowledged. First, we only evaluated exposure to outdoor air pollution without considering indoor air pollution exposure due to the lack of information about pregnant women’s activity patterns and time spent indoors. Given that both indoor and outdoor sources contribute comparably to total NO_2_ exposure in urban China, this limitation in exposure assessment may lead to non-differential exposure misclassification, potentially resulting in an underestimation of total exposure. Second, while our study found significant associations during different pregnancy stages, the timing of GDM diagnosis (typically at 24–28 weeks gestation through OGTT) may have limited our ability to fully assess the impact of third-trimester exposures, which could lead to underestimation of late-pregnancy exposure effects. Third, we relied on residential addresses to estimate pollutant exposures, which may not fully capture individual exposure variations due to mobility during pregnancy, though this should be limited as pregnancy-related relocations typically occur over short distances. Fourth, despite including comprehensive maternal characteristics, we acknowledge potential residual confounding from factors not fully captured in our study. While we collected basic socioeconomic indicators and implemented residential stability criteria, detailed information about dietary habits and physical activity patterns was limited by questionnaire design constraints. These lifestyle factors could influence both exposure patterns and GDM risk. The influence of these potential confounders has not been well established in previous studies, with two prior Socio-demographic studies reporting contrasting findings regarding the role of socioeconomic status [70,71]. Fifth, the relatively small number of GDM cases may have limited our ability to detect subtle associations, even with post-hoc power analysis. Two previous studies in Denmark examining selection bias in pregnancy cohorts showed that while participant characteristics differed between hospital-based and population-based samples, most key exposure-outcome associations remained robust. Nevertheless, caution is needed when generalizing our findings to the broader population in Wuhan [72,73]. Sixth, since our study was conducted in a single hospital, the sample may have inherent selection bias, given that women delivering at this hospital may systematically differ from the general pregnant population in Wuhan in terms of socioeconomic status and residential location.

Furthermore, there are notable uncertainties in our exposure assessment approach. Specifically, the measurement methods for air pollutants face inherent challenges in accuracy and representativeness, with potential measurement errors arising from instrument precision, calibration issues, and spatial interpolation limitations between sparse monitoring stations. These methodological constraints could lead to exposure misclassification and potentially underestimate the true associations between pollutant exposure and health outcomes. The measurement methods and uncertainties for each air pollutant monitored in this study are summarized in Table 5. For example, the gravimetric method for particulate matter is affected by environmental conditions, while gaseous pollutant measurements face various instrumental and environmental interferences [74,75]. IDW interpolation was employed to estimate pollutant concentrations at unsampled locations between monitoring stations. The accuracy of IDW interpolation is primarily influenced by two factors: the spatial distribution and density of sampling points, with more uniform and dense sampling networks yielding better results, and the selection of the power parameter, which affects the smoothness of interpolated values. A higher power parameter leads to increased smoothing that may obscure local variations, while a lower value can result in excessive fluctuations [76]. In areas with sparse monitoring coverage or uneven station distribution, the interpolation may introduce additional uncertainty into exposure estimates.

While acknowledging these methodological considerations, our study has several notable strengths. First, we utilized GLMM, which allowed us to effectively account for both fixed and random effects while exploring the complex impacts of multiple air pollutants on GDM across different pregnancy stages. This statistical approach was particularly suitable for analyzing our hierarchical data structure and examining the temporal patterns of pollutant exposure. Second, the use of IDW interpolation technique enhanced prediction accuracy by providing detailed spatial distribution of health data, supporting evidence-based decision-making in public health interventions. Third, this study was conducted in Wuhan, a major urban center with distinct seasonal pollution patterns, offering important insights into air pollution–GDM associations in rapidly developing urban environments. This geographical focus provides particularly relevant findings for similar urban settings in developing countries where air pollution remains a significant public health challenge.

Future investigations should address several critical research priorities. Prospective longitudinal cohort studies extending beyond the gestational period are essential to elucidate the potential long-term metabolic implications of prenatal air pollution exposure. The incorporation of personal exposure monitoring methodologies utilizing portable devices would facilitate more precise exposure assessment through comprehensive capture of both indoor and outdoor pollution patterns throughout pregnancy. Additionally, randomized controlled trials evaluating the efficacy of various protective interventions, including air purification systems and behavioral modification strategies, are necessary to establish evidence-based exposure reduction protocols. Furthermore, investigations examining the interactions between air pollution exposure and various modifying factors, such as dietary patterns, physical activity levels, and genetic predisposition, would enhance our understanding of susceptibility patterns within specific populations. Lastly, economic analyses evaluating the cost-effectiveness of different intervention approaches would provide valuable information for the development of targeted public health policies that aim to protect pregnant women from air pollution exposure.

## 5. Conclusions

This comprehensive analysis of air pollution exposure and GDM risk in Wuhan suggests differential temporal patterns of association across pregnancy stages. The study indicates that ${\mathrm{NO}}_{2}$ appears to be the pollutant most strongly linked to the development of GDM. Furthermore, ${\mathrm{PM}}_{2.5}$ exposure showed significant correlations during the early and mid-pregnancy periods, while ${\mathrm{SO}}_{2}$ impacts were observed primarily in the early stages of pregnancy. Sensitivity analyses conducted by stratifying populations according to urban maternity status and maternal age supported the stability of these associations. While these findings support the importance of reducing air pollution exposure during pregnancy, especially in urban areas, and suggest the potential benefit of implementing targeted interventions for susceptible populations to prevent GDM development, further validation through larger-scale studies is needed. Future research exploring the potential effects of interaction between different air pollutants may improve our understanding of these associations.

## Figures and Tables

**Table 1:** Characteristics of study participants with and without GDM.

Characteristic	GDM (n = 164)	Control (n = 731)	Total (n = 895)
**Gestational age (weeks)** ($\chi^{2}$ = 14.723, *p* = 0.023 *)			
37	6 (3.66%)	34 (4.65%)	40 (4.47%)
38	36 (21.95%)	168 (22.98%)	204 (22.79%)
39	68 (41.46%)	251 (34.34%)	319 (35.64%)
40	43 (26.22%)	197 (26.95%)	240 (26.82%)
41	4 (2.44%)	69 (9.44%)	73 (8.16%)
42	1 (0.61%)	2 (0.27%)	3 (0.34%)
<37	6 (3.66%)	10 (1.37%)	16 (1.79%)
**Maternal age (years)** ($\chi^{2}$ = 18.014, *p* = 0.001 **)			
25–29	39 (23.78%)	290 (39.67%)	329 (36.76%)
30–34	83 (50.61%)	308 (42.13%)	391 (43.69%)
35–39	32 (19.51%)	96 (13.13%)	128 (14.30%)
≥40	7 (4.27%)	16 (2.19%)	23 (2.57%)
<25	3 (1.83%)	21 (2.87%)	24 (2.68%)
**Type of residence** ($\chi^{2}$ = 0.004, *p* = 0.953)			
Rural	53 (32.32%)	238 (32.56%)	291 (32.51%)
Urban	111 (67.68%)	493 (67.44%)	604 (67.49%)
**Maternal education** ($\chi^{2}$ = 2.045, *p* = 0.360)			
Three-year college	29 (17.68%)	160 (21.89%)	189 (21.12%)
College and above	108 (65.85%)	473 (64.71%)	581 (64.92%)
Junior high school and below	27 (16.46%)	98 (13.41%)	125 (13.97%)
**Family income** ($\chi^{2}$ = 0.032, *p* = 0.858)			
≤100,000 yuan	29 (17.68%)	125 (17.10%)	154 (17.21%)
>100,000 yuan	135 (82.32%)	606 (82.90%)	741 (82.79%)
**Gravidity** ($\chi^{2}$ = 4.526, *p* = 0.210)			
1	70 (42.68%)	361 (49.38%)	431 (48.16%)
2	57 (34.76%)	199 (27.22%)	256 (28.60%)
3	21 (12.80%)	110 (15.05%)	131 (14.64%)
>3	16 (9.76%)	61 (8.34%)	77 (8.60%)
**Risk stratification in pregnancy** ($\chi^{2}$ = 55.772, *p* < 0.001 **)			
Critical risk	8 (4.88%)	28 (3.83%)	36 (4.02%)
Low risk	10 (6.10%)	261 (35.70%)	271 (30.28%)
Medium risk	146 (89.02%)	442 (60.47%)	588 (65.70%)
**Infectious Disease** ($\chi^{2}$ = 0.083, *p* = 0.773)			
Yes	3 (1.83%)	16 (2.19%)	19 (2.12%)
No	161 (98.17%)	715 (97.81%)	876 (97.88%)
**Smoking or involuntary smoking** ($\chi^{2}$ = 0.253, *p* = 0.615)			
No	151 (92.07%)	664 (90.83%)	815 (91.06%)
Yes	13 (7.93%)	67 (9.17%)	80 (8.94%)
**BMI** ($\chi^{2}$ = 360.843, *p* < 0.001 **)			
18.5–23.9	103 (62.80%)	97 (13.27%)	200 (22.35%)
Uncertain	0 (0.00%)	575 (78.66%)	575 (64.25%)
<18.5	16 (9.76%)	16 (2.19%)	32 (3.58%)
>24	45 (27.44%)	43 (5.88%)	88 (9.83%)
**Folic acid supplementation** ($\chi^{2}$ = 2.078, *p* = 0.149)			
No	11 (6.71%)	76 (10.40%)	87 (9.72%)
Yes	153 (93.29%)	655 (89.60%)	808 (90.28%)
**Natural conception** ($\chi^{2}$ = 5.089, *p* = 0.024 *)			
No	5 (3.05%)	59 (8.07%)	64 (7.15%)
Yes	159 (96.95%)	672 (91.93%)	831 (92.85%)
**Assisted reproductive technology** ($\chi^{2}$ = 5.500, *p* = 0.019 *)			
No	159 (96.95%)	670 (91.66%)	829 (92.63%)
Yes	5 (3.05%)	61 (8.34%)	66 (7.37%)
**Delivery method** ($\chi^{2}$ = 0.001, *p* = 0.969)			
Vaginal delivery	87 (53.05%)	389 (53.21%)	476 (53.18%)
Cesarean section	77 (46.95%)	342 (46.79%)	419 (46.82%)
**Infant sex** ($\chi^{2}$ = 0.067, *p* = 0.796)			
Female	74 (45.12%)	338 (46.24%)	412 (46.03%)
Male	90 (54.88%)	393 (53.76%)	483 (53.97%)
**Year** ($\chi^{2}$ = 33.251, *p* < 0.001 **)			
2019	1 (0.61%)	10 (1.37%)	11 (1.23%)
2020	56 (34.15%)	328 (44.87%)	384 (42.91%)
2021	51 (31.10%)	284 (38.85%)	335 (37.43%)
2022	56 (34.15%)	109 (14.91%)	165 (18.44%)
**Seasons** ($\chi^{2}$ = 14.194, *p* = 0.003 **)			
Winter	33 (20.12%)	206 (28.18%)	239 (26.70%)
Summer	61 (37.20%)	177 (24.21%)	238 (26.59%)
Spring	47 (28.66%)	203 (27.77%)	250 (27.93%)
Autumn	23 (14.02%)	145 (19.84%)	168 (18.77%)

* *p* < 0.05, ** *p* < 0.01.

**Table 2:** Summary of average concentrations of each air pollutant for four exposure time windows from 2017 to 2022.

Environmental Factors	Min	25th	Median	75th	Max	IQR	Mean	SD	AQG2021 ^a^
${\mathrm{PM}}_{2.5}$	6.04	24.90	33.57	49.35	156.76	24.45	39.14	20.12	15
$\left(\mu g/m^{3}\right)$									
${\mathrm{PM}}_{10}$	13.08	46.59	65.88	89.05	507.36	42.47	71.81	32.86	45
$\left(\mu g/m^{3}\right)$									
${\mathrm{SO}}_{2}$	2.23	6.35	7.68	9.52	63.06	3.18	8.30	3.12	40
$\left(\mu g/m^{3}\right)$									
${\mathrm{NO}}_{2}$	5.75	27.29	35.41	43.63	82.73	16.34	35.29	11.64	25
$\left(\mu g/m^{3}\right)$									
$O_{3}$	26.78	84.68	112.51	136.22	210.88	51.54	112.27	34.26	100 ^b^
$\left(\mu g/m^{3}\right)$									
CO	0.14	0.63	0.72	0.85	2.67	0.22	0.75	0.19	4
$\left(\mathrm{mg}/m^{3}\right)$									

^a^ AQG2021: WHO global air quality guidelines 2021. ^b^ In the AQG2021 standard, all gaseous pollutants are 24-hour averages, except for O_3_, which is a single-day maximum eight-hour average.

**Table 3:** Associations between exposure to air pollution and GDM.

Pollutant	First Trimester		Second Trimester		Third Trimester		Gestation Period	
	OR	*p*-Value	OR	*p*-Value	OR	*p*-Value	OR	*p*-Value
	(95% CI)		(95% CI)		(95% CI)		(95% CI)	
CO	0.986	0.773	0.991	0.077	1.062	0.895	1.074	0.401
	(0.961–		(0.936–		(0.959–		(0.817–	
	1.027)		1.045)		1.165)		1.330)	
NO_2_	**1.215**	**0.014**	**1.251**	**0.043**	**1.160**	**0.029**	**1.352**	**0.020**
	(1.039–		(1.031–		(1.037–		(1.067–	
	1.420)		1.472)		1.282)		1.637)	
O_3_	0.993	0.177	**1.064**	**0.022**	0.996	0.060	0.989	0.211
	(0.984–		(1.059–		(1.042–		(0.885–	
	1.003)		1.070)		1.030)		1.092)	
PM_2.5_	**1.062**	**0.012**	**1.082**	**0.014**	**1.042**	**0.042**	1.052	0.156
	(1.013–		(1.030–		(1.020–		(0.922–	
	1.112)		1.134)		1.064)		1.181)	
PM_10_	1.030	0.060	0.986	0.986	0.940	0.985	0.851	0.203
	(0.999–		(0.924–		(0.841–		(0.643–	
	1.062)		1.049)		1.039)		1.059)	
SO_2_	**1.242**	**0.001**	**1.255**	**0.010**	1.152	0.895	1.177	0.205
	(1.092–		(1.059–		(0.845–		(0.810–	
	1.412)		1.452)		1.459)		1.544)	

**Table 4:** Sensitivity analysis of associations between air pollutants and GDM risk in different populations.

Pollutant	All Maternity		Urban Maternity		Advanced Maternal Age	
	OR	*p*-Value	OR	*p*-Value	OR	*p*-Value
	(95% CI)		(95% CI)		(95% CI)	
CO	0.986	0.773	0.971	0.737	1.020	0.780
	(0.961–		(0.965–		(0.977–	
	1.027)		1.036)		1.042)	
NO_2_	**1.215**	**0.014**	**1.225**	**0.028**	**1.249**	**0.030**
	(1.039–		(1.036–		(1.034–	
	1.420)		1.433)		1.443)	
O_3_	0.993	0.177	1.021	0.149	1.011	0.165
	(0.984–		(0.999–		(1.011–	
	1.003)		1.003)		1.010)	
PM_2.5_	**1.062**	**0.012**	**1.084**	**0.003**	**1.048**	**0.032**
	(1.013–		(1.024–		(1.019–	
	1.112)		1.124)		1.137)	
PM_10_	1.030	0.060	1.025	0.095	1.038	0.145
	(0.999–		(1.003–		(1.012–	
	1.062)		1.067)		1.064)	
SO_2_	**1.242**	**0.001**	**1.265**	**0.026**	**1.291**	**0.008**
	(1.092–		(1.087–		(1.089–	
	1.412)		1.422)		1.432)	

**Table 5:** Measurement methods and uncertainty sources for air pollutants.

Pollutant	Method	Uncertainty Sources	Major Interferences
Particulate Matter (PM_10_, PM_2.5_)	Gravimetric method	- Filter conditioning (Type A) - Balance uncertainty (Type A and B) - Static charge effect - Flow rate uncertainty - Temporal/spatial scale uncertainty	- Temperature variations - Humidity effects - Volatile components - Secondary aerosol formation - Water content in high RH
SO_2_	UV fluorescence	- Zero/span drift - Linearity - Temperature effect - Light source intensity - PMT response drift - Chamber contamination	- Water vapor - Aromatic hydrocarbons - NO interference - Sample line absorption
NO_2_	Chemiluminescence	- Zero/span drift - Converter efficiency - Temperature effect - Converter aging - Reaction chamber pressure - O_3_ generator efficiency	- Water vapor - PAN - NH_3_ - Other NO_x_ interference - Chamber wall effects
O_3_	UV absorption	- Zero/span drift - Linearity - Temperature effect - Light source stability - Optical path length - Detector response	- Water vapor - Mercury vapor - Aromatic hydrocarbons - O_3_ decomposition
CO	Non-dispersive IR	- Zero/span drift - Linearity - Temperature effect - IR source stability - Detector temperature - Chamber length changes	- Water vapor - CO_2_ - Hydrocarbons - Chamber wall effects

**Note:** PMT = photomultiplier tube; UV = ultraviolet; IR = infrared; RH = relative humidity; PAN = peroxyacetyl nitrate. Type A uncertainty evaluation is based on statistical analysis of series of observations, while Type B uncertainty evaluation is based on means other than statistical analysis using available relevant information.

## Data Availability

Restrictions apply to the public availability of the study datasets. The data are available by contacting corresponding authors for collaboration or other reasonable requests with permission from the project directors, Renmin Hospital of Wuhan University, The State Key Laboratory of Information Engineering in Surveying, Mapping, and Remote Sensing, Wuhan University.

## Abbreviations

The following abbreviations are used in this manuscript:

AQG	Air Quality Guidelines
BMI	Body Mass Index
CO	Carbon Monoxide
GDM	Gestational Diabetes Mellitus
GLMM	Generalized Linear Mixed Models
hPL	human Placental Lactogen
IADPSG	International Association of Diabetes and Pregnancy Study Groups
IDW	Inverse Distance Weighting
IQR	Interquartile Range
JNK	c-Jun N-terminal Kinases
NF-κB	Nuclear Factor kappa B
NO	Nitric Oxide
NO_2_	Nitrogen Dioxide
NOx	Nitrogen Oxides
O_3_	Ozone
OGTT	Oral Glucose Tolerance Test
PAEs	Phthalate Acid Esters
PAHs	Polycyclic Aromatic Hydrocarbons
PFASs	Perfluoroalkyl Substances
PGH	Placental Growth Hormone
PM_2.5_	Particulate Matter (diameter ≤ 2.5 μm)
PM_10_	Particulate Matter (diameter ≤ 10 μm)
PTB	Preterm Birth
SCTB	Syncytiotrophoblast Cells
SD	Standard Deviation
SO_2_	Sulfur Dioxide
T2DM	Type 2 Diabetes Mellitus
TNF-α	Tumor Necrosis Factor alpha
WHO	World Health Organization
